# Diagnostic and Therapeutic Potential of Exosomes in Neurodegenerative Diseases

**DOI:** 10.3389/fnagi.2021.790863

**Published:** 2021-12-16

**Authors:** Panyue Gao, Xinrong Li, Xinzhe Du, Sha Liu, Yong Xu

**Affiliations:** ^1^Department of Psychiatry, First Hospital/First Clinical Medical College of Shanxi Medical University, Taiyuan, China; ^2^Shanxi Key Laboratory of Artificial Intelligence Assisted Diagnosis and Treatment for Mental Disorder, First Hospital of Shanxi Medical University, Taiyuan, China; ^3^Department of Mental Health, Shanxi Medical University, Taiyuan, China

**Keywords:** exosomes, neurodegenerative diseases, pathophysiology, biomarkers, treatment

## Abstract

Neurodegenerative diseases are closely related to brain function and the progression of the diseases are irreversible. Due to brain tissue being not easy to acquire, the study of the pathophysiology of neurodegenerative disorders has many limitations—lack of reliable early biomarkers and personalized treatment. At the same time, the blood-brain barrier (BBB) limits most of the drug molecules into the damaged areas of the brain, which makes a big drop in the effect of drug treatment. Exosomes, a kind of endogenous nanoscale vesicles, play a key role in cell signaling through the transmission of genetic information and proteins between cells. Because of the ability to cross the BBB, exosomes are expected to link peripheral changes to central nervous system (CNS) events as potential biomarkers, and can even be used as a therapeutic carrier to deliver molecules specifically to CNS. Here we summarize the role of exosomes in pathophysiology, diagnosis, prognosis, and treatment of some neurodegenerative diseases (Alzheimer’s Disease, Parkinson’s Disease, Huntington’s Disease, Amyotrophic Lateral Sclerosis).

## Introduction

Neurodegenerative disorders are a group of diseases closely related to brain function. Because brain tissue cannot be histologically examined, we have little understanding of its pathophysiological mechanisms, especially mental disorders. At present, the diagnosis of nervous system disease is mainly based on high-cost neuroimaging and biochemical examination of cerebrospinal fluid. Alzheimer’s Disease, Parkinson’s Disease, Amyotrophic Lateral Sclerosis and Huntington’s Disease are the important parts of the neuropsychiatric diseases. The course of neurodegenerative diseases is long and the onset is slow. Early diagnosis is difficult because of the lack of effective peripheral biomarkers (Cheng et al., 2015; Niu et al., 2020).

The blood-brain barrier (BBB) is a diffusion barrier essential for the normal functioning of CNS, preventing most molecules in the blood from entering the brain, and is composed of endothelial cells, astrocyte end-feet and pericytes (Ballabh et al., 2004). It has been shown that about 98% of small molecules and almost all macromolecules (such as peptides and gene drugs) cannot pass through the BBB, and in contrast, lipophilic small molecules pass more easily (Gabathuler, 2010). Therefore, most molecules must pass through a specific transporter protein or receptor in order to cross the BBB (Gabathuler, 2010). Previous studies have shown that the BBB is disrupted in neurodegenerative diseases, with increased permeability that is more favorable for substances to cross (Sweeney et al., 2018). However, in terms of treatment, the BBB still hinders the delivery of drugs to the CNS, making it difficult for drugs to reach the site of CNS injury because they are confined to the peripheral circulation, thus causing more drug side effects (Lakhal and Wood, 2011; Tian et al., 2018). Many researchers have attempted to increase the efficiency of drug delivery by injecting chemicals into the brain to disrupt the BBB, but this approach simultaneously disrupts the tight junctions of the BBB, leading to the invasion of harmful substances and metabolic waste, and disrupts cerebral blood flow levels, further exacerbating symptoms by affecting metabolism and neuroinflammation (Ouyang et al., 2021). How to transport therapeutic drugs more efficiently across the BBB to target sites has become a critical issue to be addressed in the treatment process.

Exosome contains DNA, RNA, proteins, lipids, and other substances, which can be circularly transmitted to adjacent and distant cells. The transfer of exosomes can lead to phenotypic changes of receptor cells (Cheng et al., 2015). In particular, miRNA is involved in a variety of pathological processes, including the growth and metastasis of tumors. Many recent studies showed that it also plays an important role in nerve inflammation (Steinbichler et al., 2017; Li et al., 2018; Pascual et al., 2020). It has been found that the production and release of exosomes depend largely on the characteristics of the mother cell and the target cell (Pegtel and Gould, 2019). The presence of some signature proteins on their surface can interact with other proteins such as integrins, which leads to the transport and fusion of exosomes, and the connection with target cells to function (Rastogi et al., 2021). Therefore, different exosomes have different contents and physiological functions with good specificity and targeting. Due to its peripheral availability and, ability to cross the BBB and superior targeting, it increases the possibility of being used as a biomarker in neurodegenerative diseases, thus manifesting its application in the diagnosis of ND (Wei et al., 2020). It can even be used as a drug carrier in the treatment of diseases (Haney et al., 2015). In conclusion, the study of exosomes in neurodegenerative diseases contributes to the early detection and diagnosis of diseases and provides new methods for the treatment of diseases.

This review summarizes the role of exosomes in some age-related neurodegenerative diseases (Alzheimer’s Disease, Parkinson’s Disease, Huntington’s Disease, Amyotrophic Lateral Sclerosis) and reveals the present the latest research progress.

## Exosome

**FIGURE 1:**
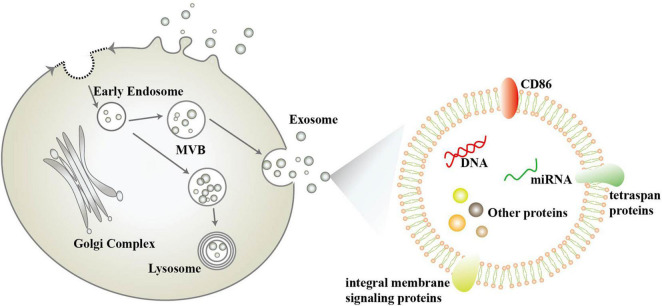
Schematic diagram of the biogenesis and composition of exosomes.

Exosomes were first described in 1981 by Trams, E. G et al. (Trams et al., 1981). They are nanoscale vesicles (30–100 nm in diameter) released by different types of cells under specific stimuli., including neurons and glial cells. In the early 1980s, the complex process of exosome generation was proposed, including the formation and release of multivesicular bodies (MVB) (Colombo et al., 2014). However, some researchers have pointed out that exosomes are not limited to one mode of generation, but can also be generated by budding of the plasma membrane (Pegtel and Gould, 2019; Figure 1). Exosomes are widely distributed in saliva, plasma, cerebrospinal fluid, milk, and other body fluids found in a variety of body fluids and contain proteins, nucleic acids, and lipids from mother cells (Geng et al., 2019; Saeedi et al., 2019; Sun et al., 2019; Figure 1). The size, shape, and density of exosomes are determined by the proteins, lipids, and other substances they contain, and are highly variable (Pegtel and Gould, 2019). Escola et al. found that co-stimulatory molecule CD86 and several tetraspan proteins (including CD37, CD53, CD63, CD81, and CD82) were highly enriched in exosomes and correlated with their immune effects (Escola et al., 1998), with CD81 and CD63 having become the most commonly used exosome marker proteins (Hemler, 2003). In addition to this, exosomes contain many integral membrane signaling proteins, including epidermal growth factor receptor (EGFR), mast/stem cell growth factor receptor (c-Kit), vascular endothelial growth factor receptor type-2, insulin-like growth factor I receptor, T cell receptor, cytokine receptors, G protein–coupled receptors (GPCRs), Notch receptors and so on, which can act as surface signaling molecules for cellular transmission of functional receptors and signaling pathways (Pegtel and Gould, 2019). In addition, the lipid content of exosomes is not the same as that of their mother cells, with cholesterol, sphingomyelin, glycosphingolipids and phosphatidylserine being enriched in exosomes two to three times as much as in their mother cells (Skotland et al., 2017).

Exosomes have an important biological role *in vivo* (Pegtel and Gould, 2019), and some researchers have categorized this into the following three points: (1) The organism can selectively remove some protein substances from the plasma membrane through the pathway of exosome generation, such as the vesicular secretion of neurodegenerative amyloidogenic proteins (Quek and Hill, 2017). (2) Exosomes are important components of the extracellular matrix that mediate amyloid aggregation, plaque and tangle formation, growth and spreading in neurodegenerative disease. (3) Exosomes mediate intercellular signaling and messaging. Exosomes can be internalized by other cells through membrane fusion, endocytosis, or specific phagocytosis. By transferring proteins and genetic materials (such as mRNA, miRNA, rRNA, long-chain non-coding RNA, DNA, etc.) to other cells, it can change the function of receptor cells and mediate cell-to-cell communication. And bilayer lipid membrane effectively protects its contents from degradation (Wang et al., 2018; Geng et al., 2019; Koteswara et al., 2019). Exosomes contain a variety of biological substances, among which miRNAs are highly enriched in exosomes. And most miRNAs that can be obtained from serum or saliva are contained in exosomes. Exosome-derived miRNAs are considered to be more stable than cell miRNAs (Wang et al., 2018). The expression profile of exosomal miRNAs is not identical to that of mother cells, and its expression can be changed according to the change of disease status. In recent years, it has been considered as a potential biomarker (Saeedi et al., 2019). More and more attention has been paid to the role of exosomes in neurodegenerative diseases (Fries and Quevedo, 2018). Especially for neurodegenerative diseases, the study of exosomal miRNAs has attracted great attention.

The ability to cross BBB (Samanta et al., 2018; Gomez-Molina et al., 2019) makes it possible for exosomes to enter the brain as a drug delivery carrier. Also, due to the targeting effect and immune resistance of exosomes (Bunggulawa et al., 2018), they can further reduce the drug side effects due to drug retention in the periphery during transport as drug carriers, further increasing the drug delivery benefits. In recent years, they are a promising drug carrier. In many types of cancer, whether *in vivo* or *in vitro*, it has been shown that they can carry drugs and target delivery to reduce the adverse reactions of drug treatment (Yang et al., 2017; Kalimuthu et al., 2018). While many preclinical studies have demonstrated the potential of exosomes to treat disease, only a handful of companies are currently conducting relevant clinical studies (Perocheau et al., 2021): Codiak Biosciences works to develop therapeutic exosomes carrying siRNAs targeting the KRAS (G12D) mutation in pancreatic cancer (Mendt et al., 2018); Avalon Globocare is developing engineered exosome therapeutics (Wang et al., 2019b).

In conclusion, exosomes, as natural carriers for transferring bioactive molecules between cells, are characterized by low immunogenicity, strong biodegradability, ability to wrap endogenous bioactive molecules, and ability to cross BBB, and are considered as a new endogenous drug delivery system (Sun et al., 2019; Zhang et al., 2019). In the nervous system, exosomes mediate cell-to-cell communication and are thought to be closely related to learning and memory, neuroinflammation, and other aspects (Wang et al., 2018; Saeedi et al., 2019).

## Exosomes and Alzheimer’s Disease

**FIGURE 2:**
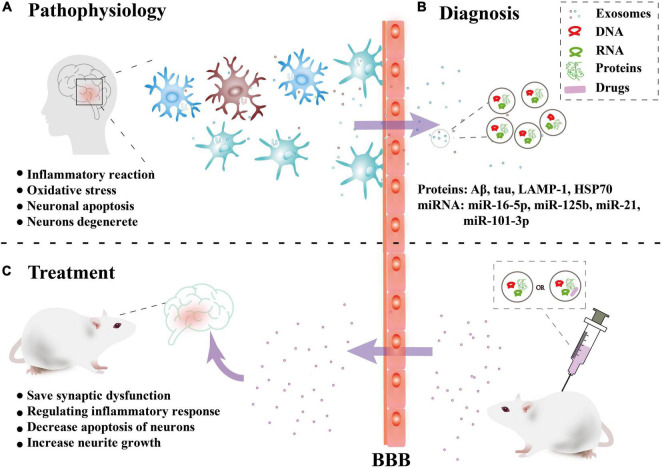
Overview of the role of exosomes in Alzheimer’s disease. **(A)** Pathophysiology: Nerve cells in brain lesions release and transmit exosomes, leading to local neuroinflammation and neuronal apoptosis, etc. **(B)** Diagnosis: Released exosomes and entering the peripheral circulation through the blood-brain barrier (BBB) were collected for diagnostic biomarkers. **(C)** Treatment: *In vitro*, exosomes combined with drugs or directly can be injected intravenously into mice can cross the BBB and have a certain targeting ability to the damaged areas, thus playing a therapeutic role.

Alzheimer’s disease (AD) is a neurodegenerative diseases characterized by decreased levels of amyloid-beta (Aβ) (reduced levels of Aβ in the cerebrospinal fluid due to deposition of Aβ in the brain), increased levels of total tau or phosphorylated tau, and a reduction in the number and function of synapses (Palop et al., 2006). The increased Aβ aggregates into soluble oligomers to activate microglia to produce an inflammatory reaction and oxidative stress. Excessive Aβ produces a cascade reaction to make neurons degenerate. The abnormal phosphorylation of tau protein can form nerve fiber tangles, which leads to a decrease in neuron function and even neuronal apoptosis (Palop et al., 2006). In recent years, a large number of studies have shown that exosomes are closely related to the occurrence and progression of AD, bringing hope for the treatment (Figure 2).

In 2006, some studies found that exosome is associated with the release of Aβ and can aggravate the brain pathology of AD by promoting the aggregation of Aβ (Rajendran et al., 2006; Dinkins et al., 2016). It can be used as a carrier to transfer toxic substances (such as phosphorylated tau protein and Aβ) between neurons (Kaur et al., 2021). Inhibiting exosome synthesis can significantly reduce the proliferation of tau protein (Wang et al., 2017; Sardar Sinha et al., 2018; Winston et al., 2019). However, the cellular origin of the active exosomes has not been determined, and both microglia and astrocytes have been proposed (Asai et al., 2015; Rosas-Hernandez et al., 2019). In addition, it has been proposed that with the progression of AD, the level of functionally specific synaptic proteins in plasma neural-derived exosomes (NDE) decreases. And synaptic proteins in NDE may be effective preclinical indicators and progression indicators of AD (Goetzl et al., 2016, 2018).

In recent years, there have been many studies on AD biomarkers in exosomes in CSF, blood, and *in vitro* cultures, mainly involving proteins and miRNAs (Table 1). In terms of proteins, HSP70 (Goetzl et al., 2015; Chanteloup et al., 2019) has received a great deal of attention in addition to the various forms of Aβ extracted in various body fluids (Watson et al., 2019). In addition, the findings of exosomal miRNAs as biomarkers are variable and lack uniform and authoritative conclusions.

**TABLE 1:** The study of exosome contents in AD biomarkers.

Molecule	Object	Source	Expression	Potential target/mechanism	Significance	References
Cathepsin D, LAMP-1, ubiquitinylated protein	Human	serum	Up	autophagic-lysosomal dysfunction	It is a pathological change that can appear 10 years before the onset of AD.	Goetzl et al., 2015
HSP70	Human	serum	It declines in the early stages of AD and is reversed in the middle and late stages.	HSP70 acts on proteins that accumulate in the brain.	May mark the extent of synaptic dysfunction or neurodegeneration.	Goetzl et al., 2015; Chanteloup et al., 2019
SNAP-25	Human	serum	Down	-	It is related to the disease progression of AD and directly reflects the characteristic of synaptic loss during the progression of AD.	Agliardi et al., 2019
miR-9-5p, miR-598	Human	CSF	Down	It plays a potential regulatory role in amyloid proteins, stress pathways, and neurotrophic signaling.	These miRNAs may be potential biomarkers for AD.	Riancho et al., 2017
miR-342-3p, miR-342-5p, miR-150-5p, miR-23b-3p, miR-29b-3p	Human	serum	Down	-	These miRNAs are collectively altered in the disease, rather than being a single biomarker.	Lugli et al., 2015
miR-124, miR-146a, miR-155, miR-21, miR-125b	-	Culture of cell	Up	These miRNAs can be transported from cell to cell *via* exosome form to mediate mRNA transcription and aggravate the inflammatory response.	MiR-21 plays an important role in signal transduction between microglial cells and neurons, especially in neuroinflammation.	Fernandes et al., 2018
hsa-miR-101-3p miR-1306, hsamiR-106b	Human	serum	Up Down	Hsa-miR-101 can target and regulate APP mRNA, thereby reducing APP level in hippocampal neurons and promoting Aβ accumulation. Hsa-miR-1306 can target to regulate APP mRNA and increase the synthesis of APP. The down-regulation of HSAMIR-106b is associated with transforming growth factor-β signaling.	Using differential miRNAs to make a random forest model for clinical classification prediction has high sensitivity and specificity.	Cheng et al., 2015
miR-125b-5p, miR-451a, miR-605-5p miR-16-5p	Human	CRF	Down in the first stages of the disease and increase in moderate and advanced stages. Down in the Young-onset AD.	Overexpression of miR-125b-5 leads to tau hyperphosphorylation and neurotoxicity and MiR-451a plays a role in neuroinflammation.	MiR-16-5p is differentially expressed in late-onset AD and Young-onset AD and maybe a special biomarker of Young-onset AD.	McKeever et al., 2018

In treatment, because exosomes can cross the BBB, its application in targeted therapy has attracted much attention. Relative to conventional AAV (adeno-associated virus), exo-AAV (AAV combine with exosomes) is more efficient at gene delivery to the brain at low vector doses. The ability of exo-AAV of evading neutralizing antibodies and transducing CNS after peripheral delivery makes it electively ingested by neurons (Hudry et al., 2016; Perets et al., 2019). In addition, some researchers combined curcumin and exosomes to inhibit tau phosphorylation and activate GSK-3/AKT pathway, to prevent neuronal death and relief symptoms (Wang et al., 2019a).

Besides, exosomes injected into the mouse models from different cells play different roles in treatment. For example:

(1) The exosomes from mesenchymal stromal cells (MSCs) or hypoxia-preconditioned MSCs could improve cognitive function (the learning and memory) by saving synaptic dysfunction and regulating inflammatory response through regulation of miR-21 (Cui et al., 2018).
(2) Treatment with exosomes from adipose-derived stem cells (ADSC-Exo) resulted in decreased Aβ42 and Aβ40 levels, increased apoptotic molecule levels (such as p53, pro-caspase-3, decreased Bcl-2 protein), and decreased apoptosis of neurons. In addition, neurite growth was also increased during the treatment (Lee et al., 2018).
(3) Exosomes from human umbilical cord mesenchymal stem cells showed the ability to repair cognitive dysfunction, help clear Aβ deposition in the brain, and reduce neuroinflammation and relief symptoms by regulating the activation of mouse brain microglia cells (Ding et al., 2018).

## Exosomes and Parkinson’s Disease

Parkinson’s disease (PD) is the second most common neurodegenerative diseases in the world after Alzheimer’s disease (Lebouvier et al., 2009). The main pathological changes were degeneration and death of dopaminergic neurons in substantia nigra, a significant decrease of DA in the striatum, and eosinophilic inclusion bodies appearance in the cytoplasm of residual neurons in substantia nigra, namely Lewy body, α-synuclein (α-syn) is its main ingredients. Recent studies have shown that exosomes play a certain role in the pathogenesis, diagnosis, and treatment of PD (Howitt and Hill, 2016).

As an important component of the Lewy bodies, α-syn are transferred from intracellular to extracellular by being packaged into exosomes or directly released (Lee, 2005). Exosomes provide an ideal environment for α-syn polymerization (Grey et al., 2015). Exosomes can transfer toxic forms of α-syn between a variety of cells, such as astrocytes and microglia, which can lead to the exacerbation of PD. α-syn deposited in glial cells induces inflammation and its further transmission promotes the degeneration of neurons and exacerbates the development of PD (Chistiakov and Chistiakov, 2017). α-syn in NDE is considered an important biomarker of PD and associated with deterioration of motor symptoms (Si et al., 2019; Niu et al., 2020). Some researchers have found that the content of α-syn in NDE of non-tremor patients is higher than that of tremor patients, so it may also be used to identify different types of motor types in PD (Si et al., 2019).

In terms of biomarkers, the value of α-syn in exosomes in diagnosis has been pointed out in many studies (Si et al., 2019; Fu et al., 2020; Niu et al., 2020; Zheng et al., 2021), and it has also been shown that exosomal α-syn can be used to differentiate PD from multiple system atrophy (Dutta et al., 2021). There are some other studies on exosomes in serum and CSF of PD patients (Table 2).

**TABLE 2:** The study of exosome contents in PD biomarkers.

Molecule	Object	Source	Expression	Potential target/mechanism	Results	References
miR-19b miR-195,miR-24	Human	serum	Down Up	Parkin RBR E3 ubiquitin protein ligase (miR-19b), LRRK2/PARK8 (miR-19b), and ATP13A2/PARK9 (miR-24 and miR-195).	ROC curve was used to evaluate the combined diagnostic value of the three miRNAs: AUC was 0.946 (95%CI, 0.910-0.981).	Cao et al., 2017
miR-153, miR-409-3p, miR-10a-5p, let-7g-3p miR-1 and miR-19b-3p	Human	CSF	Up Down	Neurotrophin signaling, mTOR signaling, Ubiquitin mediated proteolysis, Dopaminergic synapse, Glutamatergic synapse were the most prominent pathways	The sensitivity and specificity for distinguishing Parkinson’s disease from control were 94% for miR-1, 93% for miR-153, 90% for miR-409-3p, 94% for miR-19b-3p, 95% for miR-10a-5p, and 95% for let-7g-3p.	Gui et al., 2015
miR-505 miR-331-5p	Human	serum	Down Up	−	The ROC curve analysis AUC values of miRNA-331-5p and miR-505 were 0.849 and 0.898, respectively.	Yao et al., 2018
prion protein	Human	serum	Up	PrPC can increase phosphorylated α-synuclein and induce synaptic damage and calcium homeostasis.	The level of prion protein in PD plasma exosomes was significantly correlated with the level of cognitive impairment. (*t* = -3.185, *P* = 0.001)	Leng et al., 2020
DJ-1	Human	serum	Up	DJ-1 can promote disease progression by regulating α-synuclein cytotoxicity.	The ROC analysis results of DJ-1 in PD plasma neurogenic exosomes were as follows: AUC = 0.703, sensitivity = 79.5%, specificity = 57.5%.	Zhao et al., 2018

In terms of treatment, exosomes can be used as drug therapy carriers for PD and have a natural brain targeted ability. By using the blood exosomes as a delivery system, the distribution of dopamine in the brain has increased more than 15-fold. Compared with free dopamine after intravenous administration, dopamine-loaded exosomes show better therapeutic efficacy and lower systemic toxicity in a PD mouse model (Qu et al., 2018). Otherwise, the release of catalase can reduce cell death by protecting neurons from oxidative damage. *In vitro* and *in vivo* trials of PD, therapeutic catalase mRNA delivery by exosomes attenuated neurotoxicity and neuroinflammation (Haney et al., 2015; Kojima et al., 2018).

Moreover, the study on exosomes also provided a theoretical basis for other known causes of PD and clarified the specific pathological mechanism. For instance:

(1) Glucocerebrosidase gene (GBA) mutation is the most common genetic pathogenic factor in PD and is associated with decreased glucocerebrosidase activity in PD. Overexpression of GBA *in vitro* resulted in significantly reduced exosome secretion which is associated with the α-syn oligomers. And the reduction of glucocerebrosidase activity *in vivo* induced a significant increase in the number of exosomes released in the brain, thus promoting the pathological changes of PD (Papadopoulos et al., 2018).
(2) Manganese exposure is considered to be an important factor in the susceptibility of PD. It can increase the protein Rab27a and some miRNAs that regulate the release of exosomes, leading to protein aggregation, autophagy, inflammation, and hypoxia, thereby potentially promoting progressive neurodegeneration (Harischandra et al., 2018). In addition, manganese exposure promotes the secretion of α-syn in exosomes, accelerates the intercellular transmission of α-syn, and leads to dopaminergic neurotoxicity, thus causing proinflammatory and neurodegenerative reactions (Harischandra et al., 2019).
(3) Mutations in leucine-rich repetitive kinase 2 (LRRK2) enhance the level of self-phosphorylated LRRK2 protein, the most commonly known cause of hereditary PD. The level of Ser(P)-1292 LRRK2 in Urinary exosomes were found to be elevated in idiopathic Parkinson’s disease and correlated with the severity of cognitive impairment and difficulty in daily activities (Fraser et al., 2016b). Its proportion in total LRRK2 predicted LRRK2 mutation status in LRRK2 mutation carriers (Fraser et al., 2016a).

## Exosomes and Huntington’s Disease

Huntington’s disease (HD) is an autosomal dominant neurodegenerative disorder. In 1993, the international Huntington’s Disease Cooperative Research Group cloned the disease-causing gene IT15, its metabolite mutant Huntington’s protein (mHTT) which has many repeated glutamines, is easy adhesion, aggregation, and eventually lead to the death of nerve cells. Exosomes can carry and spread mHTT between cells, triggering HD-related behaviors and pathological performance (Jeon et al., 2016).

Previous studies in different HD mouse models have shown that exosomes from astrocytes (ASC-EXO) carry heat shock proteins and other neuroprotective substances, which can reduce the cytotoxicity of misfolded proteins and prevent neurodegeneration. And dysfunction of astrocytes can lead to neuronal vulnerability (Hajrasouliha et al., 2013; Nafar et al., 2016). Although mHTT does not exist in ASC-Exo, it can reduce the secretion of ASC-Exo in HD mice (Hong et al., 2017). The specific possible mechanism is as follows: (1) The n-terminal of mHTT can form aggregation in the nucleus, leading to the decrease of exosomes in astrocytes; (2) mHTT reduces the expression of the small heat shock protein αB-crystallin [a protein mainly expressed in glial cells, mediating exosome secretion (Gangalum et al., 2016)] in astrocytes, thus reducing the secretion of exosomes in the brain and promoting the acellular autonomic neurotoxicity of HD. And when ASC-Exo is injected into the striatum of HD mice, the density of mHTT aggregation could be reduced and the overexpression of αB-crystallin could reduce the exosome deficiency and neuropathological changes, which provided ideas for the treatment of Huntington’s disease (Hong et al., 2017).

It has been found that ASC-EXO could significantly reduce mHTT aggregation of nerve cells, up-regulate the expression of PGC-1 and phosphorylated CREB, and reduce mitochondrial dysfunction and cell apoptosis, indicating that ASC-EXO has the potential to treat HD (Lee et al., 2016b). In addition, it has been found that exosome-mediated hydrophobic modification of siRNA can silence HTT mRNA, which is expected to promote the development of treatment methods for Huntington’s disease and other neurodegenerative diseases (Didiot et al., 2016). Some researchers have injected exosomes loaded with excess miR-124 into the striatum of HD model mice in an attempt to improve HD-like behavior, and although the results were not satisfactory, this approach still resulted in downregulation of the target gene RE1-Silencing Transcription Factor (Lee et al., 2017). Some researchers recently found that transferring serum exosomes from young mice into an *in vitro* model of HD can effectively ameliorate mHTT mutations, slow down apoptosis, and promote mitochondrial biogenesis, while the shared blood circulation through parabiosis experiment confirmed the above idea (Lee et al., 2021).

## Exosomes and Amyotrophic Lateral Sclerosis

Amyotrophic lateral sclerosis (ALS) is caused by progressive weakness and atrophy of the muscles innervated by the medulla oblongata and muscles in the limbs and trunk after injury to upper and lower motor neurons. According to family history, it can be divided into sporadic ALS (sALS) and familial ALS (fALS), among which only 5 ∼ 10% are fALS. So far, more than 20 genes related to the pathogenesis of ALS have been found, among which superoxide dismutase gene (SODl) and ubiquitinated TAR-DNA binding protein 43(TDP43) are the most studied (Chen et al., 2019).

Some ALS are caused by the misfolding of mutated SOD1, and the misfolded SOD1 is transferred through exosome dependent or independent way. In cell culture, once SOD1 misfold happens, it can still induce misfolding of the immature cell after the initial misfolded template has been degraded for a long time (Gomes et al., 2007; Grad et al., 2014a,b; Silverman et al., 2016). Other researchers have found that NDE in the brain of ALS can cause the cytoplasmic redistribution of TDP-43, suggesting that exosomes may be involved in the transmission of TDP-43 protein lesions (Yohei et al., 2016). By analyzing serum miR-27a-3b in ALS and healthy subjects, Xu Qian et al. found that exosomal miR-27a-3b expression was down-regulated in ALS, which may serve as a detection indicator for ALS, but the exact mechanism is unclear (Xu et al., 2018).

Noriko Hayashi et al. analyzed the proteomics of exosomes from patients with sALS, it is found that three proteins increased and 11 proteins decreased in exosomes of ALS patients. The most increased protein was a new that inhibitor (NIR), which was closely related to nucleolar function. The decrease of NIR in the motor neuron nucleus of ALS patients suggested that nucleolar stress might play a role in the pathogenesis of sporadic ALS through NIR dysfunction (Hayashi et al., 2019). Chen et al. (2019) found that the level of IL-6 in ASC-Exo of sALS patients was increased, which was positively correlated with the rate of disease progression (only for patients with disease course less than 12 months), indicating that inflammation of CNS was increased, and exosomes derived from nerve cell might help to reveal the neuroinflammation of CNS of ALS patients. Besides, exosome proteins have been proved to be mainly involved in the negative regulation of cell adhesion and apoptosis. In the exosomes of ALS patients, the pro-apoptotic protein Bax and caspase-3 are down-regulated, and the antiapoptotic protein Bcl-2α is up-regulated. The protein content is related to the antiapoptotic effect of exosomes (Bonafede et al., 2019). Moreover, TDP-43 from the exosomes of patients can induce the increase of monocytes, which may increase the neuroinflammatory effect (Zondler et al., 2017). In addition, exosomes mediated the interaction mechanism between muscle and bone at the cellular level, promoted the mineralization of osteoblasts, participated in the occurrence and development of ALS, and had potential reference value for the clinical diagnosis of ALS (Xu et al., 2018).

In the treatment of ALS, ASC-Exo has been shown to save mitochondria-related dysfunction, which can reduce intracellular SOD1 aggregation, regulate the cell phenotype of ALS, and can be used as a candidate drug for ALS treatment (Lee et al., 2016a). Investigators treated an *in vitro* cell model of ALS with ASC-Exo and found that ASC-Exo rescued mitochondrial dysfunction and the mitochondrial membrane potential, thus suggesting that ASC-Exo may be used to treat diseases characterized by mitochondrial adaptations, such as ALS (Calabria et al., 2019). Subsequently, Roberta et al. tried to inject ASC-Exo into ALS mice transgenic for SOD1 (G93A) and found that this treatment significantly improved motor performance, protected lumbar motor neurons, neuromuscular junctions and muscles in ALS mice (Bonafede et al., 2020).

## Discussion

Although there are a large number of studies on exosomes in nervous system disease, mainly focusing on the pathophysiological mechanism, the disease development, biomarkers, and treatment (as carriers or themselves), in addition to the biomarker studies, most of the rest in the nervous system research is in the animal model or *in vitro* cell culture data, especially in the treatment. Applying exosomes to therapeutic safety and technical issues is a major challenge (Chen and Chopp, 2018): (1) Cell culture conditions and storage methods may have a significant impact on the contents and functions of exosomes, which requires standardization of exosome extraction methods, storage, and functional read-out systems; (2) The content, function, and activity of exosomes depend on the generating cells of origin. So, it is necessary to optimize exosome cell sources, including age, gender, comorbidities, and other factors related to the exosome-generating cells; (3) As a therapeutic method, exosomes are mainly focused on functional aspects, but the negative effects are rarely studied. In previous studies, we can see that exosomes play a certain role in promoting neurogenesis, inhibiting neuroinflammation, promoting angiogenesis, and promoting synaptic plasticity. However, in the treatment of a disease, not all the effects are meaningful for the treatment, some even bring great risks, such as whether the treatment of other non-tumor diseases will increase the risk of cancer promotion; (4) Exosome therapy is still in the preclinical trial stage. Although it can relieve symptoms, it is still a problem whether it can promote the prognosis.

In a word, exosomes bring hope for patients with neurodegenerative diseases, which has great clinical research value, provides a new method for the diagnosis and treatment of diseases, and better clarifies the pathophysiological mechanism.

## Author Contributions

PG was responsible for reviewing the literature and writing the manuscript. XL and XD participated in providing ideas for the article. SL and YX participated in revising the manuscript. All authors contributed to the article and approved the submitted version.

## Conflict of Interest

The authors declare that the research was conducted in the absence of any commercial or financial relationships that could be construed as a potential conflict of interest.

## Publisher’s Note

All claims expressed in this article are solely those of the authors and do not necessarily represent those of their affiliated organizations, or those of the publisher, the editors and the reviewers. Any product that may be evaluated in this article, or claim that may be made by its manufacturer, is not guaranteed or endorsed by the publisher.

## References

[B1] Agliardi C. Guerini F. R. Zanzottera M. Bianchi A. Nemni R. Clerici M. (2019). SNAP-25 in Serum Is Carried by Exosomes of Neuronal Origin and Is a Potential Biomarker of Alzheimer’s Disease. *Mol. Neurobiol.* 56 5792–5798. 10.1007/s12035-019-1501-x 30680692

[B2] Asai H. Ikezu S. Tsunoda S. Medalla M. Luebke J. Haydar T. (2015). Depletion of microglia and inhibition of exosome synthesis halt tau propagation. *Nat. Neurosci.* 18 1584–1593. 10.1038/nn.4132 26436904 PMC4694577

[B3] Ballabh P. Braun A. Nedergaard M. (2004). The blood-brain barrier: an overview: structure, regulation, and clinical implications. *Neurobiol. Dis.* 16 1–13. 10.1016/j.nbd.2003.12.016 15207256

[B4] Bonafede R. Brandi J. Manfredi M. Scambi I. Schiaffino L. Merigo F. (2019). The Anti-Apoptotic Effect of ASC-Exosomes in an In Vitro ALS Model and Their Proteomic Analysis. *Cells* 8:1087. 10.3390/cells8091087 31540100 PMC6770878

[B5] Bonafede R. Turano E. Scambi I. Busato A. Bontempi P. Virla F. (2020). ASC-Exosomes Ameliorate the Disease Progression in SOD1(G93A) Murine Model Underlining Their Potential Therapeutic Use in Human ALS. *Int. J. Mol. Sci.* 21:3651. 10.3390/ijms21103651 32455791 PMC7279464

[B6] Bunggulawa E. Wang W. Yin T. Wang N. Durkan C. Wang Y. (2018). Recent advancements in the use of exosomes as drug delivery systems. *J. Nanobiotechnol.* 16:81. 10.1186/s12951-018-0403-9 30326899 PMC6190562

[B7] Calabria E. Scambi I. Bonafede R. Schiaffino L. Peroni D. Potrich V. (2019). ASCs-Exosomes Recover Coupling Efficiency and Mitochondrial Membrane Potential in an in vitro Model of ALS. *Front. Neurosci.* 13:1070. 10.3389/fnins.2019.01070 31680811 PMC6811497

[B8] Cao X.-Y. Lu J.-M. Zhao Z.-Q. Li M.-C. Lu T. An X.-S. (2017). MicroRNA biomarkers of Parkinson’s disease in serum exosome-like microvesicles. *Neurosci. Lett.* 644 94–99. 10.1016/j.neulet.2017.02.045 28223160

[B9] Chanteloup G. Cordonnier M. Moreno-Ramos T. Pytel V. Matias-Guiu J. Gobbo J. (2019). Exosomal HSP70 for Monitoring of Frontotemporal Dementia and Alzheimer’s Disease: Clinical and FDG-PET Correlation. *J. Alzheimers Dis.* 71 1263–1269. 10.3233/JAD-190545 31498123

[B10] Chen J. Chopp M. (2018). Exosome Therapy for Stroke. *Stroke* 49 1083–1090. 10.1161/STROKEAHA.117.018292 29669873 PMC6028936

[B11] Chen Y. Xia K. Chen L. Fan D. (2019). Increased Interleukin-6 Levels in the Astrocyte-Derived Exosomes of Sporadic Amyotrophic Lateral Sclerosis Patients. *Front. Neurosci.* 13:574. 10.3389/fnins.2019.00574 31231184 PMC6560167

[B12] Cheng L. Doecke J. D. Sharples R. A. Villemagne V. L. Fowler C. J. Rembach A. (2015). Prognostic serum miRNA biomarkers associated with Alzheimer’s disease shows concordance with neuropsychological and neuroimaging assessment. *Mol. Psychiatry* 20 1188–1196. 10.1038/mp.2014.127 25349172

[B13] Chistiakov D. A. Chistiakov A. A. (2017). alpha-Synuclein-carrying extracellular vesicles in Parkinson’s disease: deadly transmitters. *Acta Neurol. Belg.* 117 43–51. 10.1007/s13760-016-0679-1 27473175

[B14] Colombo M. Raposo G. Théry C. J. A. R. O. C. Biology D. (2014). Biogenesis, secretion, and intercellular interactions of exosomes and other extracellular vesicles. *Annu. Rev. Cell Dev. Biol.* 30 255–289. 10.1146/annurev-cellbio-101512-122326 25288114

[B15] Cui G. H. Wu J. Mou F. F. Xie W. H. Wang F. B. Wang Q. L. (2018). Exosomes derived from hypoxia-preconditioned mesenchymal stromal cells ameliorate cognitive decline by rescuing synaptic dysfunction and regulating inflammatory responses in APP/PS1 mice. *FASEB J.* 32 654–668. 10.1096/fj.201700600R 28970251

[B16] Didiot M.-C. Hall L. M. Coles A. H. Haraszti R. A. Godinho B. M. Chase K. (2016). Exosome-mediated Delivery of Hydrophobically Modified siRNA for Huntingtin mRNA Silencing. *Mol. Ther.* 24 1836–1847. 10.1038/mt.2016.126 27506293 PMC5112038

[B17] Ding M. Shen Y. Wang P. Xie Z. Xu S. Zhu Z. (2018). Exosomes Isolated From Human Umbilical Cord Mesenchymal Stem Cells Alleviate Neuroinflammation and Reduce Amyloid-Beta Deposition by Modulating Microglial Activation in Alzheimer’s Disease. *Neurochem. Res.* 43 2165–2177. 10.1007/s11064-018-2641-5 30259257

[B18] Dinkins M. B. Enasko J. Hernandez C. Wang G. Kong J. Helwa I. (2016). Neutral Sphingomyelinase-2 Deficiency Ameliorates Alzheimer’s Disease Pathology and Improves Cognition in the 5XFAD Mouse. *J. Neurosci.* 36 8653–8667. 10.1523/JNEUROSCI.1429-16.2016 27535912 PMC4987436

[B19] Dutta S. Hornung S. Kruayatidee A. Maina K. Del Rosario I. Paul K. (2021). α-Synuclein in blood exosomes immunoprecipitated using neuronal and oligodendroglial markers distinguishes Parkinson’s disease from multiple system atrophy. *Acta Neuropathol.* 142 495–511. 10.1007/s00401-021-02324-0 33991233 PMC8357708

[B20] Goetzl E. J. Boxer A. Schwartz J. B. Abner E. L. Petersen R. C. Miller B. L. (2015). Altered lysosomal proteins in neural-derived plasma exosomes in preclinical Alzheimer disease. *Neurology* 85 40–47. 10.1212/wnl.0000000000001702 26062630 PMC4501943

[B21] Escola J. Kleijmeer M. Stoorvogel W. Griffith J. Yoshie O. Geuze H. J. (1998). Selective enrichment of tetraspan proteins on the internal vesicles of multivesicular endosomes and on exosomes secreted by human B-lymphocytes. *J. Biol. Chem.* 273 20121–20127. 10.1074/jbc.273.32.20121 9685355

[B22] Fernandes A. Ribeiro A. R. Monteiro M. Garcia G. Vaz A. R. Brites D. (2018). Secretome from SH-SY5Y APPSwe cells trigger time-dependent CHME3 microglia activation phenotypes, ultimately leading to miR-21 exosome shuttling. *Biochimie* 155 67–82. 10.1016/j.biochi.2018.05.015 29857185

[B23] Fraser K. B. Moehle M. S. Alcalay R. N. West A. B. LRRK2 Cohort Consortium. (2016a). Urinary LRRK2 phosphorylation predicts parkinsonian phenotypes in G2019S LRRK2 carriers. *Neurology* 86 994–999. 26865512 10.1212/WNL.0000000000002436 PMC4799717

[B24] Fraser K. B. Rawlins A. B. Clark R. G. Alcalay R. N. Standaert D. G. Liu N. (2016b). Ser(P)-1292 LRRK2 in urinary exosomes is elevated in idiopathic Parkinson’s disease. *Mov. Disord.* 31 1543–1550. 10.1002/mds.26686 27297049 PMC5053851

[B25] Fu Y. Jiang C. Tofaris G. Davis J. J. (2020). Facile Impedimetric Analysis of Neuronal Exosome Markers in Parkinson’s Disease Diagnostics. *Anal. Chem.* 92 13647–13651. 10.1021/acs.analchem.0c03092 32945162 PMC7584333

[B26] Gabathuler R. (2010). Approaches to transport therapeutic drugs across the blood-brain barrier to treat brain diseases. *Neurobiol. Dis.* 37 48–57. 10.1016/j.nbd.2009.07.028 19664710

[B27] Gangalum R. K. Bhat A. M. Kohan S. A. Bhat S. P. (2016). Inhibition of the Expression of the Small Heat Shock Protein alphaB-Crystallin Inhibits Exosome Secretion in Human Retinal Pigment Epithelial Cells in Culture. *J. Biol. Chem.* 291 12930–12942. 10.1074/jbc.M115.698530 27129211 PMC4933212

[B28] Goetzl E. J. Abner E. L. Jicha G. A. Kapogiannis D. Schwartz J. B. (2018). Declining levels of functionally specialized synaptic proteins in plasma neuronal exosomes with progression of Alzheimer’s disease. *FASEB J.* 32 888–893. 10.1096/fj.201700731R 29025866 PMC5888398

[B29] Goetzl E. J. Kapogiannis D. Schwartz J. B. Lobach I. V. Goetzl L. Abner E. L. (2016). Decreased synaptic proteins in neuronal exosomes of frontotemporal dementia and Alzheimer’s disease. *FASEB J.* 30 4141–4148. 10.1096/fj.201600816R 27601437 PMC5102122

[B30] Gomes C. Keller S. Altevogt P. Costa J. (2007). Evidence for secretion of Cu, Zn superoxide dismutase via exosomes from a cell model of amyotrophic lateral sclerosis. *Neurosci. Lett.* 428 43–46. 10.1016/j.neulet.2007.09.024 17942226

[B31] Gomez-Molina C. Sandoval M. Henzi R. Ramirez J. P. Varas-Godoy M. Luarte A. (2019). Small Extracellular Vesicles in Rat Serum Contain Astrocyte-Derived Protein Biomarkers of Repetitive Stress. *Int. J. Neuropsychopharmacol.* 22 232–246. 10.1093/ijnp/pyy098 30535257 PMC6403096

[B32] Fries G. R. Quevedo J. (2018). Exosomal MicroRNAs as Potential Biomarkers in Neuropsychiatric Disorders. *Methods Mol. Biol.* 1733 79–85. 10.1007/978-1-4939-7601-0_6 29435924

[B33] Grad L. I. Pokrishevsky E. Silverman J. M. Cashman N. R. J. P. (2014a). Exosome-dependent and independent mechanisms are involved in prion-like transmission of propagated Cu/Zn superoxide dismutase misfolding. *Prion* 8 331–335. 10.4161/19336896.2014.983398 25551548 PMC4601269

[B34] Grad L. I. Yerbury J. J. Turner B. J. Guest W. C. Pokrishevsky E. O’Neill M. A. (2014b). Intercellular propagated misfolding of wild-type Cu/Zn superoxide dismutase occurs via exosome-dependent and -independent mechanisms. *Proc. Natl. Acad. Sci. U. S. A.* 111 3620–3625. 10.1073/pnas.1312245111 24550511 PMC3948312

[B35] Grey M. Dunning C. J. Gaspar R. Grey C. Brundin P. Sparr E. (2015). Acceleration of alpha-synuclein aggregation by exosomes. *J. Biol. Chem.* 290 2969–2982. 10.1074/jbc.M114.585703 25425650 PMC4317028

[B36] Gui Y. Liu H. Zhang L. Lv W. Hu X. J. O. (2015). Altered microRNA profiles in cerebrospinal fluid exosome in Parkinson disease and Alzheimer disease. *Oncotarget* 6 37043–37053. 10.18632/oncotarget.6158 26497684 PMC4741914

[B37] Rosas-Hernandez H. Cuevas E. Raymick J. B. Robinson B. L. Ali S. F. Hanig J. (2019). Characterization of Serum Exosomes from a Transgenic Mouse Model of Alzheimer’s Disease. *Curr. Alzheimer Res.* 16 388–395. 10.2174/1567205016666190321155422 30907317

[B38] Hajrasouliha A. R. Jiang G. Lu Q. Lu H. Kaplan H. J. Zhang H. G. (2013). Exosomes from retinal astrocytes contain antiangiogenic components that inhibit laser-induced choroidal neovascularization. *J. Biol. Chem.* 288 28058–28067. 10.1074/jbc.M113.470765 23926109 PMC3784718

[B39] Haney M. J. Klyachko N. L. Zhao Y. Gupta R. Plotnikova E. G. He Z. (2015). Exosomes as drug delivery vehicles for Parkinson’s disease therapy. *J. Control Rel.* 207 18–30. 10.1016/j.jconrel.2015.03.033 25836593 PMC4430381

[B40] Harischandra D. S. Ghaisas S. Rokad D. Zamanian M. Jin H. Anantharam V. (2018). Environmental neurotoxicant manganese regulates exosome-mediated extracellular miRNAs in cell culture model of Parkinson’s disease: Relevance to alpha-synuclein misfolding in metal neurotoxicity. *Neurotoxicology* 64 267–277. 10.1016/j.neuro.2017.04.007 28450057 PMC5654692

[B41] Harischandra D. S. Rokad D. Neal M. L. Ghaisas S. Manne S. Sarkar S. (2019). Manganese promotes the aggregation and prion-like cell-to-cell exosomal transmission of alpha-synuclein. *Sci. Signal.* 12:eaau4543. 10.1126/scisignal.aau4543 30862700 PMC6435331

[B42] Hemler M. E. (2003). Tetraspanin proteins mediate cellular penetration, invasion, and fusion events and define a novel type of membrane microdomain. *Annu. Rev. Cell. Dev. Biol.* 19 397–422. 10.1146/annurev.cellbio.19.111301.153609 14570575

[B43] Hong Y. Zhao T. Li X. J. Li S. (2017). Mutant Huntingtin Inhibits alphaB-Crystallin Expression and Impairs Exosome Secretion from Astrocytes. *J. Neurosci.* 37 9550–9563. 10.1523/JNEUROSCI.1418-17.2017 28893927 PMC5618269

[B44] Howitt J. Hill A. F. (2016). Exosomes in the Pathology of Neurodegenerative Diseases. *J. Biol. Chem.* 291 26589–26597. 10.1074/jbc.R116.757955 27852825 PMC5207170

[B45] Hudry E. Martin C. Gandhi S. Gyorgy B. Scheffer D. I. Mu D. (2016). Exosome-associated AAV vector as a robust and convenient neuroscience tool. *Gene Ther.* 23 380–392. 10.1038/gt.2016.11 26836117 PMC4824662

[B46] Jeon I. Cicchetti F. Cisbani G. Lee S. Li E. Bae J. (2016). Human-to-mouse prion-like propagation of mutant huntingtin protein. *Acta Neuropathol.* 132 577–592. 10.1007/s00401-016-1582-9 27221146 PMC5023734

[B47] Li J. J. Wang B. Kodali M. C. Chen C. Kim E. Patters B. J. (2018). In vivo evidence for the contribution of peripheral circulating inflammatory exosomes to neuroinflammation. *J. Neuroinflam.* 15:8. 10.1186/s12974-017-1038-8 29310666 PMC5759808

[B48] Kaur S. Verma H. Dhiman M. Tell G. Gigli G. Janes F. (2021). Brain Exosomes: Friend or Foe in Alzheimer’s Disease? *Mol. Neurobiol.* 58 6610–6624. 10.1007/s12035-021-02547-y [Epub ahead of print]. 34595669

[B49] Kojima R. Bojar D. Rizzi G. Hamri C. E. El-Baba M. D. Saxena P. (2018). Designer exosomes produced by implanted cells intracerebrally deliver therapeutic cargo for Parkinson’s disease treatment. *Nat. Commun.* 9:1305. 10.1038/s41467-018-03733-8 29610454 PMC5880805

[B50] Koteswara R. Nalamolu I. Venkatesh A. (2019). Exosomes Treatment Mitigates Ischemic Brain Damage but Does Not Improve Post-Stroke Neurological Outcome. *Cell. Physiol. Biochem.* 52 1280–1291. 10.33594/000000090 31026391 PMC6996798

[B51] Rajendran L. Honsho M. Zahn T. R. Keller P. Geiger K. D. Verkade P. (2006). Alzheimer’s disease beta-amyloid peptides are released in association with exosomes. *Proc. Natl. Acad. Sci. U. S. A.* 103 11172–11177. 10.1073/pnas.0603838103 16837572 PMC1544060

[B52] Lebouvier T. Chaumette T. Paillusson S. Duyckaerts C. Bruley des Varannes S. Neunlist M. (2009). The second brain and Parkinson’s disease. *Eur. J. Neurosci.* 30 735–741. 10.1111/j.1460-9568.2009.06873.x 19712093

[B53] Lee H. J. (2005). Intravesicular Localization and Exocytosis of -Synuclein and its Aggregates. *J. Neurosci.* 25 6016–6024. 10.1523/jneurosci.0692-05.2005 15976091 PMC6724798

[B54] Lee M. Ban J. J. Kim K. Y. Jeon G. S. Im W. Sung J. J. (2016a). Adipose-derived stem cell exosomes alleviate pathology of amyotrophic lateral sclerosis in vitro. *Biochem. Biophys. Res. Commun.* 479 434–439. 10.1016/j.bbrc.2016.09.069 27641665

[B55] Lee M. Ban J. J. Yang S. Im W. Kim M. (2018). The exosome of adipose-derived stem cells reduces beta-amyloid pathology and apoptosis of neuronal cells derived from the transgenic mouse model of Alzheimer’s disease. *Brain Res.* 1691 87–93. 10.1016/j.brainres.2018.03.034 29625119

[B56] Lee M. Im W. Kim M. (2021). Exosomes as a potential messenger unit during heterochronic parabiosis for amelioration of Huntington’s disease. *Neurobiol. Dis.* 155:105374. 10.1016/j.nbd.2021.105374 33940179

[B57] Lee M. Liu T. Im W. Kim M. (2016b). Exosomes from adipose-derived stem cells ameliorate phenotype of Huntington’s disease in vitro model. *Eur. J. Neurosci.* 44 2114–2119. 10.1111/ejn.13275 27177616

[B58] Lee S. Im W. Ban J. Lee M. Jung K. Lee S. (2017). Exosome-Based Delivery of miR-124 in a Huntington’s Disease Model. *J. Mov. Disord.* 10 45–52. 10.14802/jmd.16054 28122430 PMC5288667

[B59] Leng B. Sun H. Zhao J. Liu Y. Shen T. Liu W. (2020). Plasma exosomal prion protein levels are correlated with cognitive decline in PD patients. *Neurosci. Lett.* 723:134866. 10.1016/j.neulet.2020.134866 32109555

[B60] Lugli G. Cohen A. M. Bennett D. A. Shah R. C. Fields C. J. Hernandez A. G. (2015). Plasma Exosomal miRNAs in Persons with and without Alzheimer Disease: Altered Expression and Prospects for Biomarkers. *PLoS One* 10:e0139233. 10.1371/journal.pone.0139233 26426747 PMC4591334

[B61] Pascual M. Ibáñez F. Guerri C. (2020). Exosomes as mediators of neuron-glia communication in neuroinflammation. *Neural. Regen. Res.* 15 796–801. 10.4103/1673-5374.268893 31719239 PMC6990780

[B62] McKeever P. M. Schneider R. Taghdiri F. Weichert A. Multani N. Brown R. A. (2018). MicroRNA Expression Levels Are Altered in the Cerebrospinal Fluid of Patients with Young-Onset Alzheimer’s Disease. *Mol. Neurobiol.* 55 8826–8841. 10.1007/s12035-018-1032-x 29603092 PMC6208843

[B63] Mendt M. Kamerkar S. Sugimoto H. McAndrews K. Wu C. Gagea M. (2018). Generation and testing of clinical-grade exosomes for pancreatic cancer. *JCI Insight* 3:e99263. 10.1172/jci.insight.99263 29669940 PMC5931131

[B64] Hayashi N. Doi H. Kurata Y. Kagawa H. Atobe Y. Funakoshi K. (2019). Proteomic analysis of exosome-enriched fractions derived from cerebrospinal fluid of amyotrophic lateral sclerosis patients. *Neurosci. Res.* 160 43–49. 10.1016/j.neures.2019.10.010 31669371

[B65] Nafar F. Williams J. B. Mearow K. M. (2016). Astrocytes release HspB1 in response to amyloid-beta exposure in vitro. *J. Alzheimers Dis.* 49 251–263. 10.3233/JAD-150317 26444769

[B66] Niu M. Li Y. Li G. Zhou L. Luo N. Yao M. (2020). A longitudinal study on alpha-synuclein in plasma neuronal exosomes as a biomarker for Parkinson’s disease development and progression. *Eur. J. Neurol.* 27 967–974. 10.1111/ene.14208 32150777

[B67] Ouyang Q. Meng Y. Zhou W. Tong J. Cheng Z. Zhu Q. (2021). New advances in brain-targeting nano-drug delivery systems for Alzheimer’s disease. *J. Drug Target* 1–21. 10.1080/1061186x.2021.1927055 [Epub ahead of print]. 33983096

[B68] Palop J. J. Chin J. Mucke L. (2006). A network dysfunction perspective on neurodegenerative diseases. *Nature* 443 768–773. 10.1038/nature05289 17051202

[B69] Papadopoulos V. E. Nikolopoulou G. Antoniadou I. Karachaliou A. Arianoglou G. Emmanouilidou E. (2018). Modulation of beta-glucocerebrosidase increases alpha-synuclein secretion and exosome release in mouse models of Parkinson’s disease. *Hum. Mol Genet.* 27 1696–1710. 10.1093/hmg/ddy075 29547959

[B70] Pegtel D. Gould S. J. (2019). Exosomes. *Annu. Rev. Biochem.* 88 487–514. 10.1146/annurev-biochem-013118-111902 31220978

[B71] Perets N. Betzer O. Shapira R. Brenstein S. Angel A. Sadan T. (2019). Golden Exosomes Selectively Target Brain Pathologies in Neurodegenerative and Neurodevelopmental Disorders. *Nano. Lett.* 19 3422–3431. 10.1021/acs.nanolett.8b04148 30761901

[B72] Perocheau D. Touramanidou L. Gurung S. Gissen P. Baruteau J. (2021). Clinical applications for exosomes: Are we there yet? *Br. J. Pharmacol.* 178 2375–2392. 10.1111/bph.15432 33751579 PMC8432553

[B73] Qu M. Lin Q. Huang L. Fu Y. Wang L. He S. (2018). Dopamine-loaded blood exosomes targeted to brain for better treatment of Parkinson’s disease. *J. Control Rel.* 287 156–166. 10.1016/j.jconrel.2018.08.035 30165139

[B74] Quek C. Hill A. F. (2017). The role of extracellular vesicles in neurodegenerative diseases. *Biochem. Biophys. Res. Commun.* 483 1178–1186. 10.1016/j.bbrc.2016.09.090 27659705

[B75] Rastogi S. Sharma V. Bharti P. Rani K. Modi G. Nikolajeff F. (2021). The Evolving Landscape of Exosomes in Neurodegenerative Diseases: Exosomes Characteristics and a Promising Role in Early Diagnosis. *Int. J. Mol. Sci.* 22:440. 10.3390/ijms22010440 33406804 PMC7795439

[B76] Riancho J. Vazquez-Higuera J. L. Pozueta A. Lage C. Kazimierczak M. Bravo M. (2017). MicroRNA Profile in Patients with Alzheimer’s Disease: Analysis of miR-9-5p and miR-598 in Raw and Exosome Enriched Cerebrospinal Fluid Samples. *J. Alzheimers Dis.* 57 483–491. 10.3233/JAD-161179 28269782

[B77] Kalimuthu S. Gangadaran P. Rajendran R. L. Zhu L. Oh J. M. Lee H. W. (2018). A New Approach for Loading Anticancer Drugs Into Mesenchymal Stem Cell-Derived Exosome Mimetics for Cancer Therapy. *Front. Pharmacol.* 9:1116. 10.3389/fphar.2018.01116 30319428 PMC6168623

[B78] Lakhal S. Wood M. J. (2011). Exosome nanotechnology: an emerging paradigm shift in drug delivery: exploitation of exosome nanovesicles for systemic in vivo delivery of RNAi heralds new horizons for drug delivery across biological barriers. *Bioessays* 33 737–741. 10.1002/bies.201100076 21932222

[B79] Saeedi S. Israel S. Nagy C. Turecki G. (2019). The emerging role of exosomes in mental disorders. *Transl. Psychiatry* 9:122. 10.1038/s41398-019-0459-9 30923321 PMC6438960

[B80] Samanta S. Rajasingh S. Drosos N. Zhou Z. Dawn B. Rajasingh J. (2018). Exosomes: new molecular targets of diseases. *Acta Pharmacol. Sin.* 39 501–513. 10.1038/aps.2017.162 29219950 PMC5888687

[B81] Sardar Sinha M. Ansell-Schultz A. Civitelli L. Hildesjo C. Larsson M. Lannfelt L. (2018). Alzheimer’s disease pathology propagation by exosomes containing toxic amyloid-beta oligomers. *Acta Neuropathol.* 136 41–56. 10.1007/s00401-018-1868-1 29934873 PMC6015111

[B82] Si X. Tian J. Chen Y. Yan Y. Pu J. Zhang B. (2019). Central Nervous System-Derived Exosomal Alpha-Synuclein in Serum May Be a Biomarker in Parkinson’s Disease. *Neuroscience* 413 308–316. 10.1016/j.neuroscience.2019.05.015 31102760

[B83] Silverman J. M. Fernando S. M. Grad L. I. Hill A. F. Turner B. J. Yerbury J. J. (2016). Disease Mechanisms in ALS: Misfolded SOD1 Transferred Through Exosome-Dependent and Exosome-Independent Pathways. *Cell Mol. Neurobiol.* 36 377–381. 10.1007/s10571-015-0294-3 26908139 PMC11482315

[B84] Skotland T. Sandvig K. Llorente A. (2017). Lipids in exosomes: Current knowledge and the way forward. *Prog. Lipid Res.* 66 30–41. 10.1016/j.plipres.2017.03.001 28342835

[B85] Sun X. Jung J. H. Arvola O. Santoso M. R. Giffard R. G. Yang P. C. (2019). Stem Cell-Derived Exosomes Protect Astrocyte Cultures From in vitro Ischemia and Decrease Injury as Post-stroke Intravenous Therapy. *Front. Cell. Neurosci.* 13:394. 10.3389/fncel.2019.00394 31551712 PMC6733914

[B86] Sweeney M. Sagare A. Zlokovic B. V. (2018). Blood-brain barrier breakdown in Alzheimer disease and other neurodegenerative disorders. *Nat. Rev. Neurol.* 14 133–150. 10.1038/nrneurol.2017.188 29377008 PMC5829048

[B87] Steinbichler T. B. Dudás J. Riechelmann H. Skvortsova I. I. (2017). The role of exosomes in cancer metastasis. *Semin. Cancer Biol.* 44 170–181. 10.1016/j.semcancer.2017.02.006 28215970

[B88] Tian T. Zhang H. X. He C. P. Fan S. Zhu Y. L. Qi C. (2018). Surface functionalized exosomes as targeted drug delivery vehicles for cerebral ischemia therapy. *Biomaterials* 150 137–149. 10.1016/j.biomaterials.2017.10.012 29040874

[B89] Trams E. Lauter C. Salem N. Heine U. (1981). Exfoliation of membrane ecto-enzymes in the form of micro-vesicles. *Biochim. Biophys. Acta* 645 63–70. 10.1016/0005-2736(81)90512-5 6266476

[B90] Geng W. Tang H. Luo S. Lv Y. Liang D. Kang X. (2019). Exosomes from miRNA-126-modified ADSCs promotes functional recovery after stroke in rats by improving neurogenesis and suppressing microglia activation. *Am. J. Transl. Res.* 11 780–792. 30899379 PMC6413259

[B91] Wang H. Sui H. Zheng Y. Jiang Y. Shi Y. Liang J. (2019a). Curcumin-primed exosomes potently ameliorate cognitive function in AD mice by inhibiting hyperphosphorylation of the Tau protein through the AKT/GSK-3beta pathway. *Nanoscale* 11 7481–7496. 10.1039/c9nr01255a 30942233

[B92] Wang L. Yin P. Wang J. Wang Y. Sun Z. Zhou Y. (2019b). Delivery of mesenchymal stem cells-derived extracellular vesicles with enriched miR-185 inhibits progression of OPMD. *Artif Cells Nanomed. Biotechnol.* 47 2481–2491. 10.1080/21691401.2019.1623232 31219352

[B93] Wang W. Li D. B. Li R. Y. Zhou X. Yu D. J. Lan X. Y. (2018). Diagnosis of Hyperacute and Acute Ischaemic Stroke: The Potential Utility of Exosomal MicroRNA-21-5p and MicroRNA-30a-5p. *Cerebrovasc. Dis.* 45 204–212. 10.1159/000488365 29627835

[B94] Wang Y. Balaji V. Kaniyappan S. Kruger L. Irsen S. Tepper K. (2017). The release and trans-synaptic transmission of Tau via exosomes. *Mol. Neurodegener.* 12:5. 10.1186/s13024-016-0143-y 28086931 PMC5237256

[B95] Watson L. Hamlett E. Stone T. Sims-Robinson C. J. M. (2019). Neuronally derived extracellular vesicles: an emerging tool for understanding Alzheimer’s disease. *Mol. Neurodegener.* 14:22. 10.1186/s13024-019-0317-5 31182115 PMC6558712

[B96] Wei Z. X. Xie G. J. Mao X. Zou X. P. Liao Y. J. Liu Q. S. (2020). Exosomes from patients with major depression cause depressive-like behaviors in mice with involvement of miR-139-5p-regulated neurogenesis. *Neuropsychopharmacology* 45 1050–1058. 10.1038/s41386-020-0622-2 31986519 PMC7162931

[B97] Winston C. N. Aulston B. Rockenstein E. M. Adame A. Prikhodko O. Dave K. N. (2019). Neuronal Exosome-Derived Human Tau is Toxic to Recipient Mouse Neurons in vivo. *J. Alzheimers Dis.* 67 541–553. 10.3233/JAD-180776 30584143 PMC8373009

[B98] Xu Q. Zhao Y. Zhou X. Luan J. Cui Y. Han J. (2018). Comparison of the extraction and determination of serum exosome and miRNA in serum and the detection of miR-27a-3p in serum exosome of ALS patients. *Intractable Rare Dis. Res.* 7 13–18. 10.5582/irdr.2017.01091 29552440 PMC5849619

[B99] Yao Y. F. Gao Q. Li J. L. Xue C. X. Fang W. Jing J. H. (2018). Circulating exosomal miRNAs as diagnostic biomarkers in Parkinson’s disease. *Eur. Rev. Med. Pharmacol. Sci.* 22 5278–5283. 30178852 10.26355/eurrev_201808_15727

[B100] Yang J. Zhang X. Chen X. Wang L. Yang G. (2017). Exosome Mediated Delivery of miR-124 Promotes Neurogenesis after Ischemia. *Mol. Ther. Nucleic Acids* 7 278–287. 10.1016/j.omtn.2017.04.010 28624203 PMC5415550

[B101] Yohei I. Lara E. Martin P. Geneviève S. Christine B. Yuichi R. (2016). Exosome secretion is a key pathway for clearance of pathological TDP-43. *Brain* 139 3187–3201. 10.1093/brain/aww237 27679482 PMC5840881

[B102] Zhang H. Wu J. Wu J. Fan Q. Zhou J. Wu J. (2019). Exosome-mediated targeted delivery of miR-210 for angiogenic therapy after cerebral ischemia in mice. *J. Nanobiotechnol.* 17 29. 10.1186/s12951-019-0461-7 30782171 PMC6379944

[B103] Zhao Z. H. Chen Z. T. Zhou R. L. Zhang X. Ye Q. Y. Wang Y. Z. (2018). Increased DJ-1 and alpha-Synuclein in Plasma Neural-Derived Exosomes as Potential Markers for Parkinson’s Disease. *Front. Aging Neurosci.* 10:438. 10.3389/fnagi.2018.00438 30692923 PMC6339871

[B104] Zheng H. Xie Z. Zhang X. Mao J. Wang M. Wei S. (2021). Investigation of α-Synuclein Species in Plasma Exosomes and the Oligomeric and Phosphorylated α-Synuclein as Potential Peripheral Biomarker of Parkinson’s Disease. *Neuroscience* 469 79–90. 10.1016/j.neuroscience.2021.06.033 34186110

[B105] Zondler L. Feiler M. S. Freischmidt A. Ruf W. P. Ludolph A. C. Danzer K. M. (2017). Impaired activation of ALS monocytes by exosomes. *Immunol. Cell Biol.* 95 207–214. 10.1038/icb.2016.89 27616750

